# N-acetylcysteine in the Management of Acute Exacerbation of Chronic Obstructive Pulmonary Disease

**DOI:** 10.7759/cureus.6073

**Published:** 2019-11-05

**Authors:** Sheeba F Ansari, Mubeen Memon, Naveed Brohi, Amber Tahir

**Affiliations:** 1 Internal Medicine, Liaquat University of Medical and Health Sciences, Jamshoro, PAK; 2 Pulmonology, Civil Hospital, Jamshoro, PAK; 3 Pulmonology, Jinnah Postgraduate Medical Centre Hospital, Karachi, PAK; 4 Internal Medicine, Dow University of Health Sciences, Karachi, PAK

**Keywords:** acute exacerbation of copd, pakistan, n-acetyl cysteine (nac), aecopd, acute exacerbations, chronic obstructive pulmonary disease

## Abstract

Introduction

Chronic obstructive pulmonary disease (COPD) is a preventable disease of the airways characterized by limited airflow. Acute exacerbations of COPD (AECOPD) may be precipitated by noxious stimuli. N-acetylcysteine (NAC) has mucolytic, antioxidant, and anti-inflammatory activity. We conducted this study to evaluate the effect of adding high-dose NAC to the protocol treatment of AECOPD.

Methods

In this single-center, prospective, interventional study, patients admitted with AECOPD, airflow obstruction on spirometry, and who were current smokers with 10 or more packs per year were included after attaining informed consent. NAC granules 600 mg twice daily orally (high dose) were included in the regimen of 25 randomly selected patients and the other 25 were managed without NAC. An improvement in clinical and biochemical markers was observed on day three and day seven. For statistical analysis, SPSS for Windows version 21.0 (IBM Corp., Armonk, NY) was utilized.

Results

The study was completed by 21 patients in the NAC group and 19 in the non-NAC group. In the NAC group, there was a significant improvement in the mean partial pressure of oxygen (PaO_2_) both on day three (p=0.03) and day seven (p=0.01). The mean partial pressure of carbon dioxide (PaCO_2_) was at the borderline in the two groups on day three; however, on day seven, the NAC group showed significantly improved PaCO_2_ as compared to the non-NAC group (p=0.007). There were significant improvements in oxygen saturation of the NAC group on day seven (p=0.02). There were significant improvements in clinical signs, including wheezing and dyspnea and the need for nasal oxygen support (p≤0.05).

Conclusion

The addition of 600 mg twice daily NAC (high dose) to the protocol treatment of patients with acute exacerbation of COPD may have beneficial outcomes. In the future, the role of high-dose NAC in AECOPD must be studied through multicenter, double-blinded, placebo-controlled trials with larger sample sizes in order to either establish or invalidate this association.

## Introduction

Chronic obstructive pulmonary disease (COPD) is a common, treatable, and preventable disease of the airways that is characterized by limited airflow. It is triggered by an inflammatory response to continuous exposure of the airways to noxious gases or particles (such as in smokers) and presents with persistent airway symptoms [1]. COPD is the third leading cause of death worldwide [2].

In the Asia-Pacific region, the prevalence of COPD is 6% with a profound impact on the quality of life of these patients. Two-fifth of these patients have work restriction, one-fifth have severe persistent airway symptoms, and one-fifth required hospital admissions for acute exacerbations of COPD (AECOPD) [3]. AECOPD may be precipitated by smoking, air pollution, or other noxious stimuli. As COPD progresses, acute attacks become more frequent, have a higher recurrence, and may be life-threatening [4].

Global Initiative for Chronic Obstructive Lung Disease (GOLD) in their 2019 recommendations indicated that mucolytic/antioxidant therapy with N-acetylcysteine (NAC), carbocysteine, and other mucoregulators may help in improving the overall health status and prevent acute exacerbations [5]. Since 2015, a few notable trials have been conducted to evaluate the role of NAC in both AECOPD prevention and management. Placebo-controlled study on efficacy and safety of N-acetylcysteine high dose in exacerbations of chronic obstructive pulmonary disease (PANTHEON study) provided some controversial evidence regarding concomitant inhaled corticosteroid therapy and smoking on the protective effect of NAC in AECOPD prevention [6]. On the other hand, in a recent meta-analysis, it was deduced that only the quality of randomized controlled trials (RCT), treatment duration, and the frequency of AECOPD in the past can have a significant impact on the efficacy of NAC in AECOPD prevention [7].

In a double-blind, placebo-controlled RCT, NAC was introduced with protocol treatment in patients with AECOPD, which did not modify the outcome [8]. Aytemur et al. hypothesized that in acute exacerbations, NAC may only exert its mucolytic effects, hence, they included a subset of COPD patients with increased sputum production; however, they failed to find any promising outcome [9]. In another double-blind, placebo-controlled RCT, NAC successfully normalized C-reactive protein (CRP) levels, interleukin-8 (IL-8), and improved expectoration in patients with AECOPD [10]. In view of this contradictory evidence, we conducted this study to evaluate the role of NAC in AECOPD along with protocol treatment.

## Materials and methods

It was a single-center, prospective, interventional study conducted with in-hospital patients of AECOPD. The study was conducted in the pulmonology department of Civil Hospital, Pakistan, from October 1, 2018, till December 31, 2018. The study was conducted after approval from the institutional review board.

In this study, the inclusion criteria comprised patients of both genders, of any age, admitted in the ward with acute exacerbation of COPD, airflow obstruction on spirometry, and who were current smokers with 10 or more packs per year. Patients who had concomitant asthma, bronchiectasis, evidence of pneumonia on chest X-ray, survival-limiting comorbidity (e.g., metastatic malignancy), or were critical enough to require intensive care unit (ICU) admission and/or mechanical ventilation were excluded from the study. Patients who had taken any mucolytic drug in the preceding week were also excluded. Informed written consent was attained from all patients.

All patients were given protocol treatment for AECOPD, which included inhaled anticholinergics and beta-agonists, intravenous antibiotics, and systemic steroids as determined by their pulmonologists. Twenty-five patients were randomized to receive NAC granules 600 mg twice daily orally (high-dose NAC) and the other 25 received only the protocol treatment. The primary endpoint of the study was an improvement in the biochemical and clinical profile of the patients. The biochemical parameters included in the study were oxygen saturation, partial pressure of oxygen (PaO_2_), and partial pressure of carbon dioxide (PaCO_2_). The clinical characteristics included wheezing and dyspnea. All parameters were measured thrice during the study - once at the start, second at day three of intervention, and third at day seven or discharge day, whichever came first. As per hospital protocol, patients of AECOPD are admitted for seven days at least for monitoring and stabilization.

For statistical analysis, SPSS for Windows version 21.0 (IBM Corp., Armonk, NY) was utilized. Mean and standard deviation (SD) were calculated for continuous variables. The independent T-test was used to compare these variables for the NAC and non-NAC groups. For categorical variables, frequencies and percentages were calculated and chi-square was applied for a comparison between the two groups.

## Results

The study was completed by 21 patients in the NAC group and 19 in the non-NAC group. The mean age of the study sample was 46.2 ± 10.7 years; patients in the NAC group were slightly younger. There were 26 (65%) men and 14 (35%) women in the study. Both the NAC and non-NAC groups had more men than women. The mean duration of COPD was 10.6 ± 2.1 years; patients in the NAC group suffered from COPD for a slightly longer duration. In both groups, 38%-47% of patients had been admitted more than once for AECOPD in the past year.

The mean PaO_2_, PaCO_2_, and oxygen saturation were compared for both groups at the start of the study. The demographic, clinical, and biochemical parameters of the patients at the start of the study are compared in Table 1.

**Table 1:** Baseline characteristics of the study participants at the start of the study (N=40) Abbreviations: AECOPD, Acute Exacerbation of Chronic Obstructive Pulmonary Disease; COPD, Chronic Obstructive Pulmonary Disease; M/F, Male / Female; NAC, N-acetylcysteine; PaO_2_, Partial Pressure of Oxygen; PaCO_2_, Partial Pressure of Carbon Dioxide

Baseline Characteristics	NAC group (n=21)	Non-NAC group (n=19)	P-value
Mean age, years	43.6 ± 11.5	47.1 ± 9.8	0.31
Gender (M/F)	15/6	11/8	0.37
Duration of COPD, years	9.8 ± 3.9	9.3 ± 2.6	0.63
≥1 hospital admissions for AECOPD in past the year, %	8 (38.1%)	9 (47.4%)	0.55
Mean PaO_2_ level, mmHg	53.7 ± 13.4	56.8 ± 12.6	0.45
Mean PaCO_2_ level, mmHg	46.5 ± 11.5	48.1 ± 14.6	0.70
Mean oxygen saturation, %	84.3 ± 6.3	85.5 ± 5.7	0.53
Wheeze, %	20 (95.2%)	16 (84.2%)	0.24
Dyspnea at rest, %	17 (80.9%)	14 (73.7%)	0.58
Dyspnea at night, %	12 (57.1%)	12 (63.2%)	0.68
Dyspnea only on exertion, %	2 (9.5%)	4 (21%)	0.30

After NAC administration in one group along with protocol treatment and only protocol treatment in the other group, their clinical and biochemical characteristics were compared on day three and day seven of intervention. In the NAC group, there was a significant improvement in the mean PaO_2_ both on day three (p=0.03) and on day seven (p=0.01). PaCO_2_ was borderline normal in the two groups on day three; however, on day seven, the NAC group showed significantly improved PaCO_2_ as compared to the non-NAC group (p=0.007). There were significant improvements in oxygen saturation of the NAC group on day seven (p=0.02). There were significant improvements in clinical signs, including wheezing and dyspnea, and the need for nasal oxygen support. All clinical and biochemical characteristics of the NAC and non-NAC groups on day three and seven are compared in Table 2.

**Table 2:** Change in the clinical and biochemical characteristics of the patients on day three and day seven of N-acetylcysteine administration Abbreviations: NAC, N-acetylcysteine; NA, Not Applicable; PaO_2_, Partial Pressure of Oxygen; PaCO_2_, Partial Pressure of Carbondioxide

Patient Characteristics	On Day 3 of Admission			On Day 7 of Admission		
	NAC group (n=21)	Non-NAC group (n=19)	P value	NAC group (n=21)	Non-NAC group (n=19)	P value
Mean PaO_2_ level, mmHg	79.4 ± 15.3	70.3 ± 10.7	0.03	93.5 ± 3.5	90.1 ± 4.8	0.01
Mean PaCO_2_ level, mmHg	45.1 ± 4.2	46.3 ± 4.6	0.39	37.1 ± 3.2	39.8 ± 2.8	0.007
Mean oxygen saturation, %	92.4 ± 4.3	89.7 ± 5.1	0.07	97.6 ± 1.2	96.1 ± 2.7	0.02
Wheeze, %	4 (19%)	10 (52.6%)	0.02	1 (4.8%)	6 (31.6%)	0.02
Dyspnea at rest, %	5 (23.8%)	7 (36.8%)	0.36	0	2 (10.5%)	NA
Dyspnea at night, %	1 (4.8%)	0	NA	0	1 (5.3%)	NA
Dyspnea only on exertion, %	3 (14.3%)	9 (47.4%)	0.02	1 (4.8%)	4 (21%)	0.11
Need for nasal oxygen support, %	6 (28.5%)	12 (63.2%)	0.03	3 (14.3%)	9 (47.4%)	0.02

## Discussion

In this study, there was a significant improvement in the biochemical and clinical parameters of patients who were given low-dose NAC (600mg/day) as compared to those who were managed without NAC.

N-acetylcysteine has two major roles in alleviating inflammation in AECOP. It acts as an antioxidant and a mucolytic agent and has anti-inflammatory actions [8]. Its antioxidant actions are because it acts as a reduced glutathione precursor, which is a direct antioxidant as well as a substrate of other antioxidants [11-12]. The role of free oxygen radicals in the precipitation of an acute attack of COPD has been previously established, which was evident by the increased exhalation of hydrogen peroxide during an exacerbation episode [13]. NAC also breaks down thiolated proteins, releasing free thiols and other reduced proteins that have direct antioxidant activity. NAC also breaks down disulfide, which is responsible for its mucolytic activity [11-12]. Severe and frequent COPD exacerbations have been associated with sputum production and chronic cough [14]. The anti-inflammatory effects of NAC in AECOPD have been studied in Zuin et al. where high-dose NAC helped in alleviating CRP and IL-8 levels, both of which are inflammatory markers [10].

There have been little data to study the impact of NAC in acute exacerbations of COPD. When Zuin et al. compared low-dose NAC (600 mg/day), high-dose NAC (1200 mg/day), and placebo, only high-dose NAC produced substantial results [10]. In our study, high-dose NAC was given, which can be a reason for our results being different as compared to the previous literature. This may be reinforced by the fact that Black et al. also compared low-dose NAC (600 mg/day) with placebo in their RCT [8]. Their continued NAC for seven days in their patients admitted with AECOPD and did not find any significant improvement in terms of dyspnea, forced expiratory volume in one second (FEV_1_), oxygen saturation, and duration of hospital stay. They mentioned low-dose NAC and small sample size (n=50) as the limitations of their work [8].

The beneficial role of NAC in the long-term prevention of AECOPD has been studied before [6] and owing to its strong evidence, it has been included in the GOLD 2019 document [5]. However, the role of NAC in AECOPD remains controversial. This study has contributed to the existing controversy regarding the role of NAC in the acute management of COPD. We successfully showed the positive effects of high-dose NAC in AECOPD patients. However, it has its limitations too. It was not placebo-controlled and some airway parameters couldn’t be included in the study. Furthermore, serum CRP and other inflammatory markers were not evaluated, hence, this study cannot comment on the antioxidant role of NAC. We recommend more robust trials with high-dose NAC in AECOPD patients to strengthen or invalidate this association. Multicenter, double-blinded, placebo-controlled trials with a larger sample size, including a more heterogeneous group, will provide substantial results in this field of medical research.

## Conclusions

The addition of 600 mg twice daily NAC (high dose) to the protocol treatment of patients with acute exacerbation of COPD may have beneficial outcomes in terms of wheezing, breathlessness, partial pressures of oxygen and carbon dioxide, and oxygen saturation. However, due to the small sample size, the results of this study cannot be generalized. In the future, the role of high-dose NAC in AECOPD must be studied through stringent trials in order to either establish or invalidate this association.
